# Clonal myelopoiesis promotes adverse outcomes in chronic kidney disease

**DOI:** 10.1038/s41375-021-01382-3

**Published:** 2021-08-19

**Authors:** Ahmed A. Z. Dawoud, Rodney D. Gilbert, William J. Tapper, Nicholas C. P. Cross

**Affiliations:** 1 Faculty of Medicine, University of Southampton, Southampton, UK; 2 Southampton Children’s Hospital, Southampton, UK; 3 Wessex Regional Genetics Laboratory, Salisbury NHS Foundation Trust, Salisbury, UK

**Keywords:** Haematological diseases, Cancer genomics

## Abstract

We sought to determine the relationship between age-related clonal hematopoiesis (CH) and chronic kidney disease (CKD). CH, defined as mosaic chromosome abnormalities (mCA) and/or driver mutations was identified in 5449 (2.9%) eligible UK Biobank participants (*n* = 190,487 median age = 58 years). CH was negatively associated with glomerular filtration rate estimated from cystatin-C (eGFR.cys; *β* = −0.75, *P* = 2.37 × 10^–4^), but not with eGFR estimated from creatinine, and was specifically associated with CKD defined by eGFR.cys < 60 (OR = 1.02, *P* = 8.44 × 10^–8^). In participants without prevalent myeloid neoplasms, eGFR.cys was associated with myeloid mCA (*n* = 148, *β* = −3.36, *P* = 0.01) and somatic driver mutations (*n* = 3241, *β* = −1.08, *P* = 6.25 × 10^–5^) associated with myeloid neoplasia (myeloid CH), specifically mutations in *CBL*, *TET2*, *JAK2*, *PPM1D* and *GNB1* but not *DNMT3A* or *ASXL1*. In participants with no history of cardiovascular disease or myeloid neoplasms, myeloid CH increased the risk of adverse outcomes in CKD (HR = 1.6, *P* = 0.002) compared to those without myeloid CH. Mendelian randomisation analysis provided suggestive evidence for a causal relationship between CH and CKD (*P* = 0.03). We conclude that CH, and specifically myeloid CH, is associated with CKD defined by eGFR.cys. Myeloid CH promotes adverse outcomes in CKD, highlighting the importance of the interaction between intrinsic and extrinsic factors to define the health risk associated with CH.

## Introduction

Clonal hematopoiesis (CH) is an age-related phenomenon characterised by a gradual replacement of polyclonal leucocytes by one or more clones marked by somatic mutations [1, 2] or mosaic chromosomal alterations (mCA) [3, 4]. CH is associated with an elevated relative risk of developing haematological malignancies compared to age and sex-matched controls without CH [5], and also an elevated risk of developing non-malignant, immune and inflammatory disorders [6, 7] such as atherosclerotic cardiovascular disease (CVD) [2, 8], chronic obstructive pulmonary disease [9] and premature menopause [10].

Chronic kidney disease (CKD) is a common worldwide health problem defined by low estimated glomerular filtration rate (eGFR) and/or elevated urine albumin to creatinine ratio (uACR) [11]. Patients with CKD experience a gradual and progressive loss of kidney function, but only small minority progress to end-stage kidney disease (ESKD) and require kidney replacement therapy. The majority of cases are at an early stage of the disease process [12], which remains incompletely defined due to variation in eGFR and albuminuria measurements [13–17].

Like CH, CKD is associated with an elevated risk of CVD and mortality [18]. Atherosclerotic risk factors for CVD, such as diabetes, smoking, hypertension and dyslipidemia, are prevalent in individuals with CKD, but there is an excess risk of CVD associated with CKD that is over and above that captured by atherosclerotic risk factors alone. In addition to sharing some risk factors, CH, CKD and CVD are characterised by persistent low-grade inflammation [19–22]. however, a specific relationship between CH and CKD has not been defined. In this study, we sought to assess the relationship between CH and CKD in UK Biobank (UKB), an ongoing, prospective UK cohort study of approximately 500,000 community-dwelling participants aged 40–69 years when recruited between 2006 and 2010.

## Methods

### Study cohort

UKB participants provided comprehensive demographic, psychosocial and medical information during an initial assessment along with baseline blood and urine samples for genomic, biochemical, and other laboratory tests. Long-term follow-up was provided via linked medical records [23]. We focused on participants with both genome wide single nucleotide polymorphism (SNP) array and whole exome sequence (WES) data (*n* = 200,361; median age = 58 years, median follow up = 11 years) [24]. All participants provided informed consent according to the Declaration of Helsinki; UKB received ethical approval from the North West multi-centre Research Ethics Committee (REC reference 11/NW/0382).

### Identification of CH

We previously described the identification of myeloid, lymphoid or other mCA in UKB from SNP array data [25]. The process for identifying likely somatic driver variants is described in the Supplementary Methods. Mutated genes were defined as myeloid-neoplasia related (‘myeloid’) according to previously published criteria [8], other genes were defined as ‘lymphoid’. The complete list of unique putative somatic driver variants (*n* = 1611) is shown in Supplementary Table 1. CH was defined as participants with any mCA and/or any somatic driver mutation; myeloid CH was defined as the presence of myeloid mCA and/or a myeloid somatic driver mutation(s); lymphoid was defined by lymphoid mCA and/or lymphoid mutations, without myeloid mutations or myeloid mCA [8].

### Kidney function

The eGFR in units of mL/min/1.73 m^2^ was calculated in R using the Nephro package [26] and three different formulae as defined by the Chronic Kidney Disease Epidemiology Collaboration: creatinine (UKB field: 30700, eGFR.creat), cystatin-C (UKB field: 30720, eGFR.cys) or creatinine and cystatin-C (eGFR.creat.cys) [27]. The creatinine-based scores included ethnicity as recorded in UKB field: 21000. With respect to CKD, patients were considered as healthy (≥90), mild (≥60 and <90), moderate (≥15 and <60) or end stage (<15) for each eGFR score [27]. In addition, uACR in mg/mmol was calculated as a further measure of kidney disease using albumin in urine (UKB field: 30,500) and creatinine in urine (UKB field: 30,510). Shrunken pore syndrome (SPS) is typically defined by an eGFR.cys/eGFR.creat ratio of ≤0.6 in the absence of factors that interfere with cystatin C or creatinine measurement, such as high muscle mass [28]. We defined potential SPS as an eGFR.cys/eGFR.creat ratio of ≤0.6.

### Discovery and validation cohorts

To investigate the relationship between CKD and either CH or myeloid neoplasia, the data were split randomly into equally sized discovery and validation cohorts. Results from the discovery and validation cohorts were combined using a fixed effect inverse variance weighted meta-analysis using STATA version 16 (StataCorp LLC, College Station, TX) and Cochran’s *Q* test to measure heterogeneity.

### The relationship between CH and CKD

To study the association between CH and CKD we excluded 10,144 participants due to (i) missing creatinine or cystatin-C data (*n* = 9913), (ii) any form of ESKD (*n* = 231) that was either diagnosed before study entry according to relevant ICD10 codes or interventions and procedures as detailed in the Supplementary Methods, or if any of the three eGFR scores was <15. Participants with ESKD were excluded due to the possibility of dialysis and/or erythropoietin treatment that would influence their eGFR scores and blood counts, and because the relationship between ESKD and CVD is well characterised. Individuals with diabetes or hypertension were not excluded. The relationship between CH and CKD was tested using multivariable logistic regression in R where CKD was used as the dependant and CH as a binary predictor. CKD was coded into cases (1) and controls (0) using the eGFR thresholds of <60 or ≥60, respectively [29] and the analysis was repeated for each eGFR score (eGFR.creat, eGFR.cys, and eGFR.creat.cys). Logistic regressions were adjusted for potential confounding variables: age, sex, smoking status, systolic blood pressure, diastolic blood pressure, cholesterol, high-density lipoprotein (HDL), low-density lipoprotein (LDL), body mass index (BMI), glycated haemoglobin (HbA1c) as an indicator of diabetes, high-sensitivity C-reactive protein (hs-CRP) as a marker of inflammation and the first 10 genetic principal components. Effect sizes were reported as odds ratios (OR) with 95% confidence intervals (CI). The relationship between eGFR scores and CH was tested using multivariable linear regression in R where eGFR status was treated as the dependant and CH as a binary predictor and correcting for same confounding variables. UKB did not include follow-up biochemical assessments for the great majority of participants and so incident ESKD was inferred from recorded hospital episodes as indicated above. Prevalent and incident myeloid neoplasia are defined in the Supplementary Methods.

### Mendelian randomisation (MR)

MR was used to assess the possibility of a causal relationship between CH and CKD by using germline SNPs associated with the development of CH as instrumental variables. Following the STROBE guidelines [30], we investigated the use of two significance thresholds for selecting instrumental variables based on their association with CH defined by driver somatic mutations in a subset of the TOPMed cohort (*n* = 65,405 total participants; *n* = 3831 CHIP cases) [31]. The first used a modest threshold (*P* < 0.001) to select 380 SNPs with MAF ≥ 0.01 and SNPs clumped (r^2^ > 0.001, within 10 Mb) for a liberal analysis which aimed to investigate the evidence for a true null relationship. In the second, conservative, analysis we used a stricter threshold (*P* < 1 × 10^–5^) to select a subset of 28 SNPs that were strongly associated with CH and would provide more robust evidence of causality. The effect sizes on CKD were obtained from a meta-analysis of 60 GWAS from the CKDgene consortium (*n* = 625,219, including 64,164 CKD cases) [32]. We estimated that ~2.4% of individuals from the TOPMed cohort are also included in the CKDgene consortium which could inflate false-positive findings [33]. To mitigate against this, we performed a sensitivity analysis using the estimated effect sizes in a subset of patients from the CKDgene cohort with European ancestry (*n* = 480,698, including 41,395 cases). Detailed information for the SNPs used in both analyses is shown in Supplementary Table 2. MR was performed using the TwoSamplesMR package in R [34] to apply the Robust Adjusted Profile Score (MR-RAPS) methodology which enables the use of weak instrumental variables, is robust to pleiotropy and considers measurement error in the exposure estimate [35]. Additional sensitivity analyses were performed using methods that test the different assumptions of MR, specifically the inverse-variance weighted (IVW) method which performs a meta-analysis for the estimates of the instrumental variants [36], the MR-Egger method which uses the average pleiotropic effect as the intercept that allow the use of instrumental variables with pleiotropic effects [37], and the weighted median method which allows for a subset of instrumental variables to be invalid [38].

### Prediction of adverse outcomes

A Cox proportional hazard model (survival package in R) [39] was used to determine if the risk of adverse outcome was associated with CH, CKD defined by each eGFR score or the urine albumin-to-creatinine ratio (uACR). Adverse outcomes were defined by a composite endpoint of either death (UKB data release April 2020), myocardial infarction (MI, field 40002, February 2018) or stroke (field 40006, February 2018). Participants who suffered MI or stroke before entering UKB were excluded. Follow-up times were calculated using the lubridate package [40] to determine the duration between study entry and the earliest of date of death (UKB field 40000), date of MI (UKB field 40002) or date of stroke (UKB field 40006). Patients without an adverse outcome were censored at the date of last follow-up for MI and stroke or the date of they were lost to follow-up (UKB field 191). Univariate survival analyses were performed for all traditional risk factors (age, sex, smoking status, LDL, HDL, cholesterol, HbA1c, BMI, hs-CRP, systolic and diastolic blood pressure). Variables with *P* < 0.2 were entered into a multivariate survival analysis in a backward stepwise manner and retained if they reached nominal significance (*P* < 0.05).

To assess the potential for a non-linear relationship between eGFR scores and adverse outcomes, we used a restricted cubic spline function [41] to transform and segment the eGFR scores. Separate curves were fitted to each segment to generate a smooth fitted curve. The method was used to transform each eGFR score using the rms package in R [42] and default values for the number of knots (*n* = 5) and degrees of freedom (*n* = 4). The regression included the covariates described above. The adjusted spline values were plotted with 95% CI.

Receiver operating characteristic curves (ROC) and area under the curve (AUC) metrics [43] were used to evaluate the prediction accuracy of the multivariable survival models. AUCs were reported for three pairs of prediction models with and without CH: (i) traditional risk factors, (ii) traditional risk factors and eGFR.cys and (iii) traditional risk factors and uACR. Where relevant, *P* values for all tests were corrected for multiple testing using the false discovery rate (FDR).

## Results

### Definition and breakdown on CH in UKB

In a previous analysis of SNP array data from the entire UKB cohort, we identified 8203 mCA larger than 2 Mb in 5040 participants [44]. In the subset of participants with available WES data (*n* = 200,631), we identified 3085 mCA in 2016 participants, of which 197 (185 participants) were associated with myeloid neoplasms and 278 (237 participants) were associated with lymphoid neoplasms. Analysis of the WES data identified 4137 putative somatic driver mutations (1611 unique variants) in 3863 participants (Supplementary Table 3). In total, 5718 (2.9%) participants had CH defined by one or more mCA and/or driver mutations and 194,913 participants were considered as CH-free controls. For further analysis, these data were split randomly into discovery and validation cohorts (Table 1 and Supplementary Table 4).

**Table 1:** CH defined by both acquired mCA and/or driver somatic mutations.

Participants	Discovery cohort					Validation cohort					Total
	Males		Female		Total	Males		Females		Total	
	*N*	%	*N*	%		*N*	%	*N*	%		
Total number	45,198	45	55,118	55	100,316	44,956	45	55,359	55	100,315	200,631
All CH	1286	45	1582	55	2868	1335	47	1515	53	2850	5718
Myeloid CH^a^	831	46	960	54	1791	885	49	912	51	1797	3588
Lymphoid CH^b^	135	48	146	52	281	140	46	167	54	307	588
All mCA	431	42	585	58	1016	439	44	561	56	1000	2016
Myeloid mCA	48	53	42	47	90	54	57	41	43	95	185
Lymphoid mCA	57	50	56	50	113	60	48	64	52	124	237
Other mCA	326	40	487	60	813	325	42	456	58	781	1594
All driver mutations	894	47	1027	53	1921	941	48	1001	52	1942	3863
Myeloid genes^c,d^	805	46	933	54	1738	854	49	890	51	1744	3482
*DNMT3A*	295	39	470	61	765	348	45	419	55	767	1532
*TET2*	193	46	225	54	418	188	46	217	54	405	823
*ASXL1*	103	64	59	36	162	92	64	51	36	143	305
*JAK2*	37	58	27	42	64	46	57	35	43	81	145
Other myeloid genes	220	54	190	46	410	225	52	207	48	432	842
Lymphoid genes	89	49	94	51	183	87	44	111	56	198	381
Control (CH-free)	43,912	45	53,536	55	97,448	43,621	45	53,844	55	97,465	194,913

^a^79 participants had both myeloid mutations and myeloid mCA.

^b^Lymphoid CH was defined by lymphoid mCA and/or lymphoid mutations, without myeloid mutations or myeloid mCA. 30 participants had both lymphoid mutations and lymphoid mCA.

^**c**^14 participants had both myeloid and lymphoid mutations and were classed as myeloid.

^d^218 participants had more than one myeloid gene mutation.

### Assessment of the relationship between CH and CKD

**Fig. 1:**
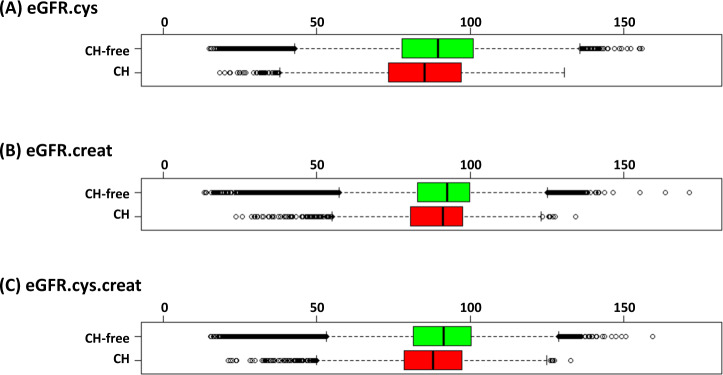
CH is associated with lower eGFR scores. Meta-analysis of discovery and validation cohorts (cases with CH, *n* = 5449; controls without CH, *n* = 185,038). **A** eGFR.cys: CH, median = 84.4; CH-free, median = 88.6 (*P* < 0.001; Mann–Whitney test), **B** eGFR.creat: CH median = 88.7; CH-free, median = 90.7 (*P* < 0.001), **C** eGFR.creat.cys: CH, median = 87.2; CH-free, median = 90.4 (*P* < 0.001).

We compared eGFR.cys, eGFR.creat and eGFR.creat.cys in participants with or without CH after excluding 10,144 ineligible participants with pre-existing ESKD or missing biochemistry measures. After excluding ineligible cases, the discovery cohort consisted of 2735 participants with CH and 92,457 CH-free controls, and the validation cohort compromised of 2714 participants with CH and 92,581 CH-free controls. As expected, the cystatin-C-derived eGFR score was lower than the scores that included creatine [45] and consequently fewer participants were determined to have moderate CKD, defined by an eGFR score between 15 and 60, according to eGFR.creat (*n* = 4194) and eGFR.creat.cys (*n* = 4433) compared with eGFR.cys (*n* = 8304). The median for all three eGFR scores was lower in participants with CH compared to those without CH (Fig. 1) and the median uACR was higher (1.2 with CH versus 1.05 without CH; *P* < 0.001) indicating impairment of kidney function in association with CH. Participants with lower eGFR scores tended to be older, male, smokers, with low HDL, high LDL, high BMI, high systolic and diastolic blood pressure, and high albuminuria (Supplementary Table 4).

**Fig. 2:**
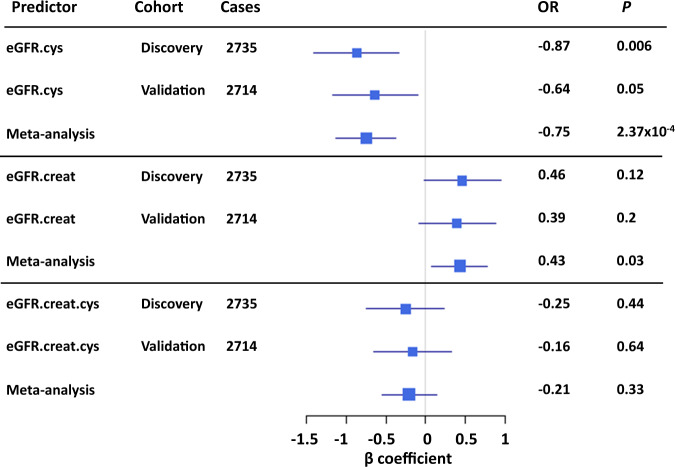
CH is specifically and negatively associated with eGFR estimated from cystatin-C. eGFR.cys: eGFR estimated from cystatin-C, eGFR.creat: eGFR estimated from creatinine, eGFR.creat.cys: estimated from both creatinine and cystatin-C. Square sizes represent the precision of each eGFR score.

To determine the association between CH and CKD, we performed logistic and linear regression analyses where CKD was coded as either a binary (1 = moderate CKD eGFR > 15 and <60, 0 = eGFR ≥60) or as a continuous trait based on each eGFR score and adjusted for potential confounding variables (Supplementary Table 5). In the logistic models, CH was associated with an increased risk of moderate CKD estimated from cystatin-C scores (eGFR.cys, OR = 1.02 [95% CI: 1.01–1.02], *P* = 8.44 × 10^−8^). A weaker association was observed for eGFR.creat.cys (OR = 1.01 [95% CI: 1.00–1.01], *P* = 0.04) and there was no association with eGFR.creat (OR = 1.00 [95% CI: 0.995–1.004], *P* = 0.93) (Supplementary Table 6). Similar results were obtained from linear regression analysis where eGFR scores estimated from cystatin-C were negatively associated with CH (eGFR.cys, *β* = −0.75, *P* = 2.37 × 10^−4^) but not eGFR.creat.cys (*β* = −0.21, *P* = 0.33), or eGFR.creat (*β* = 0.43, *P* = 0.03, not significant in the discovery and validation cohorts) (Fig. 2). For all tests there was no evidence for heterogeneity between the discovery and validation cohorts (*P* > 0.05, Cochran’s *Q* test).

To investigate the relationship between CH and CKD in more detail, we tested the constituent components of CH for association with eGFR.cys as a continuous trait using linear regression. Low eGFR.cys scores were associated with myeloid mCA (*β* = −4.44, *P* = 8.90 × 10^–5^) but not lymphoid mCA (*β* = −1.7, *P* = 0.12) or other mCA (*β* = 0.61, *P* = 0.15). Alterations involving chr9p were the most strongly associated subtype of myeloid mCA (*β* = −8.06, *P* = 8.80 × 10^−5^). For CH defined by somatic mutations, myeloid neoplasia-associated genes were strongly associated with lower levels of eGFR.cys (*β* = −1.33, *P* = 5.52 × 10^−7^), whereas lymphoid genes were not significant (*β* = −1.31, *P* = 0.125). At the gene level, the relationship was significant for CH defined by *JAK2* (*n* = 139, *β* = −1.03, *P* < 1 × 10^–300^) and *TET2* (*n* = 788, *β* = −1.94, *P* = 4.50 × 10^–4^) variants but not *DNMT3A* or *ASXL1*. Again, for all tests there was no evidence for heterogeneity between the discovery and validation cohorts (*P* > 0.05, Cochran’s *Q* test). Full results for the discovery and validation cohorts are presented in Supplementary Table 7. The median VAF of CH defined by myeloid neoplasia associated genes was higher in participants with CKD (eGFR < 60) defined by eGFR.cys (median VAF = 0.24) compared to other participants (eGFR ≥ 60) (median VAF = 0.21, *P* = 1.71 × 10^–7^) but no difference was seen for CKD defined by eGFR.creat (median VAF = 0.23 vs. 0.21, *P* = 0.12) (Supplementary Fig. 1). At the level of individual genes, a significant difference was only seen for *JAK2* with a median VAF of 0.56 in cases with CKD defined by eGFR.cys compared to other participants (VAF = 0.20, *P* = 4.70 × 10^–6^).

The link between myeloid neoplasms and reduced kidney function is well established and was replicated in our subset of UKB participants which included 320 participants with a prevalent myeloid neoplasms (diagnosed before or within a year of study entry) that was associated with lower eGFR.cys score (*β* = −5.22, *P* = 7.77 × 10^–10^). Excluding these cases, eGFR.cys was still associated with myeloid CH (*n* = 3,330, *β* = −1.05, *P* = 8.80 × 10^–5^), including both myeloid mCA (*n* = 148, *β* = −3.36, *P* = 0.01) and myeloid related-genes (*n* = 3241, *β* = −1.08, *P* = 6.25 × 10^–5^). Stratification at the gene level identified associations between eGFR.cys and mutations in *CBL*, *TET2*, *JAK2*, *PPM1D* and, to a lesser degree, *GNB1* (Table 2; Supplementary Table 8).

**Table 2:** Association between myeloid CH subtypes and eGFR.cys score after removing participants with prevalent myeloid neoplasms.

Predictor	Cases	*β*	CI 2.5%	CI 97.5%	*P*
myeloid CH	3330	−1.05	−1.54	−0.57	8.80 × 10^–5^
myeloid mCA	148	−3.36	−5.65	−1.07	9.11 × 10^–3^
myeloid genes	3241	−1.08	−1.57	−0.60	6.25 × 10^–5^
*DNMT3A*	1446	0.14	−0.59	0.87	0.73
*TET2*	778	−1.74	−2.74	−0.73	1.84 × 10^–3^
*ASXL1*	283	−1.59	−3.21	0.03	0.09
*JAK2*	92	−4.69	−7.56	−1.82	3.21 × 10^–3^
*GNB1*	86	−3.51	−6.40	−0.62	0.04
*SRSF2*	67	−2.42	−5.94	1.11	0.27
*TP53*	62	−0.01	−3.30	3.29	1.00
*PPM1D*	61	−5.87	−9.43	−2.31	3.08 × 10^–3^
*SF3B1*	52	−2.37	−6.27	1.52	0.32
*FLT3*	36	−0.65	−5.49	4.19	0.81
*GNAS*	33	1.89	−2.61	6.40	0.49
*NF1*	28	−1.03	−6.07	4.01	0.73
*CBL*	28	−12.14	−17.40	−6.88	3.44 × 10^–5^
*STAG2*	27	3.90	−1.37	9.16	0.23
*PRPF40B*	26	2.96	−2.29	8.21	0.35
*CREBBP*	24	−3.10	−8.49	2.30	0.34
*KDM6A*	22	−2.68	−8.49	3.14	0.45
*BRCC3*	21	−1.72	−7.70	4.27	0.66
*IDH2*	14	−5.56	−13.78	2.67	0.28
*KMT2D*	13	−3.43	−10.54	3.69	0.44

59 participants had both myeloid mutated genes and mCA.

We assessed the relationship between myeloid CH and the risk of developing ESKD in participants without prevalent myeloid neoplasms or prior ESKD. Myeloid CH (*n* = 3330) was weakly but significantly associated with ESKD incidence (*n* = 307, *β* = 0.002, *P* = 0.006). Specifically, 0.33% (11 out of 3330) of participants with myeloid CH developed ESKD after study entry compared with 0.16% of controls (296 of 184,811).

### MR analysis to test causal effect of CH on kidney function

**Fig. 3:**
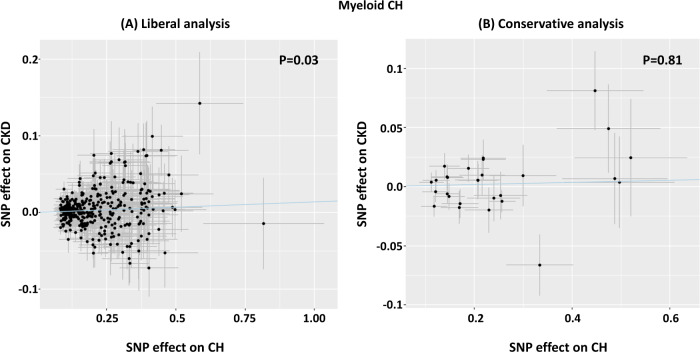
Mendelian randomisation using robust adjusted profile score (MR-RAPS) to estimate the effect of SNPs associated with CH against their effect in relation to CKD. **A** Liberal analysis using 380 independent SNPs associated with CH at *P* < 0.001. The MR-RAPS test estimated a significant positive effect of CH on CKD (OR = 1.014, CI 95%:1.003–1.024; *P* = 0.03). **B** Conservative analysis using 28 SNPs associated with CH at *P* < 1 × 10^−5^. The line of regression is indicated in blue and the axes show β coefficients for SNP effects on CH and CKD.

The possibility of a causal relationship between CH and kidney function was assessed using MR. In a liberal analysis, 380 independent SNPs associated with CH at (*P* < 0.001) [31] were used to estimate the effect of CH on CKD (Supplementary Table 2). To test the different assumptions and scenarios, several MR methods were used as recommended and the results corrected for multiple testing [33]. Only the MR-RAPS method, which is adapted to test weak instrumental variables as applicable to our study, identified a positive causal relationship [OR = 1.01; *P* = 0.029]. However, this relationship failed to reach significance (*P* = 0.81) in a more conservative analysis that applied stricter threshold (*P* < 1 × 10^–5^) to select 28 SNPs associated with CH (Fig. 3). Due to the potential limited overlap between cohorts used to select instrumental variables, we performed a sensitivity analysis using a subset of samples with European American ancestry which yielded similar results for the causal association between CH and CKD [OR = 1.02; *P* = 0.029]. Detailed results are presented in Supplementary Table 9.

### Prediction of adverse outcomes by myeloid CH in CKD

As expected, established risk factors (myeloid CH, age, sex, ethnicity, smoking status, cholesterol, HbA1C, HDL, LDL, blood pressure, BMI, uACR, hs-CRP and eGFR scores) were associated on univariate analysis with an adverse outcome as defined by a composite endpoint of death, MI, or stroke (Supplementary Table 10).

To understand the influence of myeloid CH and CKD on adverse outcomes, we focused on participants without prevalent myeloid neoplasms (*n* = 320) or any prior history of CVD (*n* = 8459). Initially, Cox proportional-hazard analysis was used to identify risk factors unrelated to CH and CKD (Supplementary Table 11), and then these factors were added into the model. To determine which of the three eGFR scores was most appropriate to use in the model, we tested the linearity of each score in relation to outcome using a restricted cubic spline test, as described previously [45]. Although all three scores were associated with adverse outcomes, eGFR.cys was more linear and negative compared to the scores that used creatinine in both the discovery and validation cohorts (Supplementary Fig. 2). Focusing on eGFR.cys, the risk of adverse outcomes was higher in subjects who had CKD (HR = 1.9, *n* = 1180/6970) compared to CKD free participants (*n* = 8295/172,857; *P* = 8.4 × 10^–65^) (Supplementary Table 12). The risk of adverse outcomes was estimated to be 1.56-fold higher (*P* = 1.4 × 10^–11^) in cases with myeloid CH (*n* = 338/3078) compared to myeloid CH-free participants (*n* = 9137/176,749). Testing each component of adverse outcomes confirmed the previously reported features of UKB cohort [46] that CH was associated with all-cause mortality (HR = 1.91, *P* = 2.5 × 10^–10^) but did not reach significance for MI (HR = 1.13, *P* = 0.38) or stroke (HR = 1.28, *P* = 0.15) considered independently, in accordance with previous findings [44, 47] (Supplementary Table 12).

**Fig. 4:**
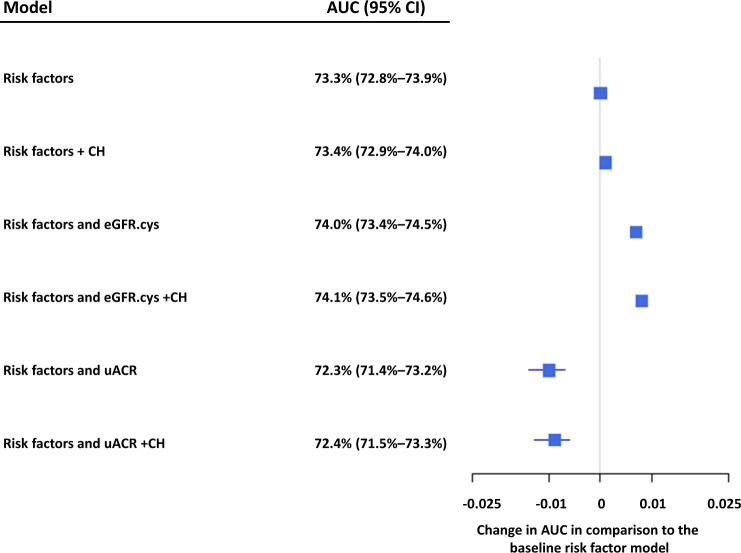
Risk factors for adverse outcome. The baseline risk factors included age, sex, smoking status, HDL, HbA1c, systolic blood pressure, hs-CRP, BMI and was corrected for 10 genetic principal components. The effect on AUC of adding in CH, eGFR.cys, and uACR relative to the baseline model is shown (meta-analysis of discovery and validation cohorts).

ROC analysis was used to assess the predictiveness of multivariable models that incorporated myeloid CH, eGFR.cys and uACR. The baseline model consisting of age, sex, smoking status, HDL, HbA1c, systolic blood pressure, hs-CRP, BMI (Supplementary Table 12) and corrected for 10 genetic principal components had an AUC of 73.3% (72.8–73.9%). The addition of myeloid CH as a binary factor or eGFR.cys as a continuous trait improved the predictiveness of the model to an AUC of 73.4% and 74%, respectively, and including both further improved the AUC to 74.1% (73.5–74.6%), with very similar results achieved in both the discovery and validation cohorts (Fig. 4, Supplementary Table 13).

**Fig. 5:**
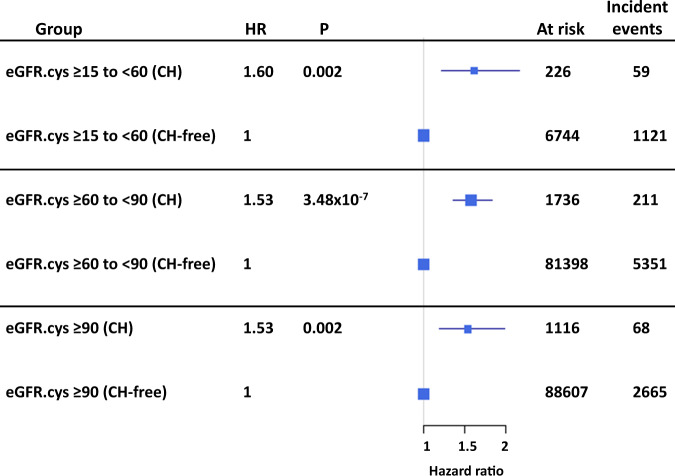
Myeloid CH predicts adverse outcomes in CKD. The forest plots show data stratified according to eGFR.cys as healthy (≥90), mild CKD (≥60 to <90) and moderate CKD (≥15 to <60). The risk of adverse outcomes was predicted by myeloid CH in all groups but was particularly marked (HR = 1.6, *P* = 0.002) for participants with moderate CKD.

**Fig. 6:**
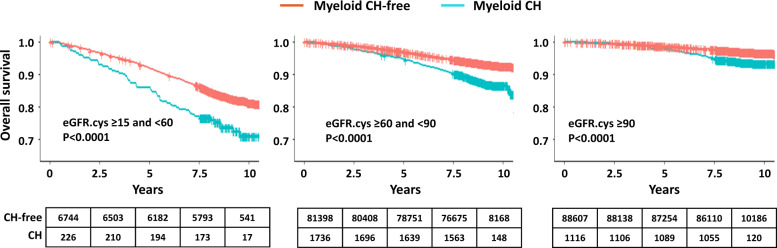
Kaplan–Meier survival estimates for the three CKD groups according to absence or presence of myeloid CH. Log-rank test *P* values are reported for each group, and numbers at risk at 0, 2.5, 5, 7.5, and 10 years after study entry.

To further investigate the relationship between CH and adverse outcome in participants with CKD, we stratified the cohort (excluding prior CVD and prevalent myeloid malignancies), into participants with moderate renal impairment (eGFR.cys ≥15 to <60), mild impairment (eGFR.cys ≥60 to <90) and normal kidney function (eGFR.cys ≥90). We then tested the effect of CH in each subset using Kaplan–Meier survival analysis. CH increased the risk of adverse outcome in all groups but was particularly marked (HR = 1.6, 95% CI 1.2–2.14, *P* = 0.002) for participants with moderate CKD (*n* = 59/226 with myeloid CH compared to *n* = 1121/6744 without myeloid CH) (Figs. 5 and 6; Supplementary Table 14). Much of the risk of adverse outcomes was related to incident myeloid neoplasms which were diagnosed in 19 participants at a median of 3.6 years after study entry. Of these, 11 (58%) had adverse outcomes in comparison to 48/207 (23%) who did not develop a myeloid neoplasm during the study period. Excluding the incident cases reduced but did not eliminate the risk of adverse outcomes (HR = 1.4, *P* = 0.05).

### Relationship between myeloid CH and SPS

We identified 966 (0.5%) UKB participants with potential SPS (eGFR.cys/eGFR.creat ratio ≤0.6). Of these, 58 (6.0%) had myeloid CH compared to 2.9% (*n* = 5391) of participants with eGFR.cys/eGFR.creat ratio > 0.6 (OR = 2.2, 95% CI = 1.6–2.9; *P* = 2.9 × 10^–7^ Fisher’s exact test), but after eliminating these cases myeloid CH was still associated with an adverse prognosis in CKD (HR = 1.61, 95% CI 1.17–2.21, *P* = 0.003) and remained most pronounced for participants with moderate renal impairment (Supplementary Fig. 3).

## Discussion

In this study we identified that CH, and specifically myeloid CH, is associated with CKD. The association was not seen with all markers of CH and, strikingly, not with mutations in *DNMT3A* or *ASXL1*, two of the most common drivers of clonality, although there was an overall association with clone size. These findings confirm previous observations that that not all CH is equal [9, 25, 31], as well as the importance of having sufficiently large studies to understand the granularity of CH with respect to clinical outcomes.

We found that myeloid CH is specifically associated with eGFR.cys but not eGFR.creat and only marginally with eGFR.cys.creat. Similarly, recent studies have reported the superior utility of eGFR.cys in predicting the incidence of CVD and mortality in patients with CKD [45, 48, 49]. In the UK, the cost to measure cystatin C is 10-fold higher than that to measure serum creatinine, and consequently eGFR.creat is widely used for initial assessment of possible CKD. Although eGFR.cys is recommended to confirm CKD, this is not believed to be common practice, at least in the UK [45]. Our findings provide further weight to the argument that eGFR.cys is more informative than eGFR.creat to define CKD.

The finding that myeloid CH is associated with eGFR.cys also provides further evidence for the importance of chronic inflammation in CH-related disorders. Levels of cystatin C correlate generally with oxidative stress and inflammation [45, 50], a well-recognized feature of CKD [19] that is also associated with an elevated risk of development of CVD [51, 52]. Other biomarkers of chronic inflammation have been associated with CH, e.g. C-reactive protein and IL-6. [20, 31] CH predisposes to haematological malignancies, particularly myeloid neoplasms [5], and both CKD and chronic inflammation have been described as features of myeloproliferative neoplasms [53, 54]. Our data show that myeloid CH increases the risk of adverse outcomes in the context of CKD and that this increase is only partly explained by incident myeloid neoplasms or SPS, a recently described phenomenon that may be observed in both children or adults with normal or reduced eGFR and is associated with increased mortality and morbidity in a variety of settings [28]. Although our analysis was corrected for hs-CRP, it is possible that part of the increase in adverse outcomes is due to chronic inflammation induced by CH.

MR uses genetic variation as a natural experiment to estimate causality in observational data [33] and has, for example, been used to detect a causal effect of cystatin C on risk of stroke [55]. Our initial analysis of 380 SNPs that predispose to CH provided suggestive evidence for a causal relationship between CH and CKD (*P* = 0.03), but this link was not supported by a more conservative analysis of 28 SNPs that are more strongly associated with CH. Given that two of the most common CH genes (*DNMT3A* and *ASXL1*) were not associated with CKD, and that the 380 SNPs only explain 3.6% of the heritability of CH [31], the use of MR in this context is clearly challenging, and may be compounded the possibility of other factors such as horizontal pleiotropy but these concerns are partly mitigated by the large sample size of the GWAS used for CH and CKD.

In summary, the role of CH in the pathogenesis of benign diseases varies widely and depends on intrinsic factors that define the clone as well as extrinsic factors that impact the inflammatory environment [25, 56]. In this study, we have shown that CH is associated with CKD and confers an adverse prognosis over and above conventional risk factors for this common disorder. Our findings suggest that screening for CH in CKD may be of clinical value to help predict outcomes.

## Supplementary information

Supplementary Methods and Figures

Supplementary Tables
