# Supplementary material for: Clonal myelopoiesis promotes adverse outcomes in chronic kidney disease
Source: Leukemia. 2021 Aug 19;36(2):507–15. doi: 10.1038/s41375-021-01382-3 (PMC8807385; doi:10.1038/s41375-021-01382-3)
Supplement: Supplementary file 1 — Supplementary Methods and Figures [file 41375_2021_1382_MOESM1_ESM.pdf]

Supplementary Methods and Figures

Clonal myelopoiesis promotes adverse outcomes in chronic kidney disease

Ahmed A.Z. Dawoud, Rodney D. Gilbert, William J Tapper & Nicholas C.P. Cross

Supplementary Methods

Identification of clonal hematopoiesis from WES data

To identify putative somatic driver mutations from WES data, individual gVCF files from the DeepVariant version 0.10.0 caller[1] were converted to VCF format and merged into one multi-sample VCF using SAMtools/Bcftools[2]. Multi-allelic variants were split into separate variants and the location of indels was normalised using their left most position.[3] The multisample VCF was annotated using Annovar and the RefSeq gene database.[4] Variants were defined as putative somatic driver mutations if they met the following criteria; (i) exonic, or splice donor/acceptor site; (ii) the alternative allele had a minimum of 3 reads for point mutation and 6 reads for indels; (iii) alternate allele frequency ≤1% in GnomAD V2.1;[5] (iv) predicted to be pathogenic (CADD[6] phred score >20 meaning that the variant is among the 1% most deleterious variants in the human genome); (v) minor allele frequency (MAF) ≤ 0.01% in UKB; (vi) observed in COSMIC version 91 database[7] at least 3 times in hematopoietic and lymphoid tissues; (vii) inferred as somatic by failing the hypothesis that the alternative allele is normally distributed with a mean of 0.45 and a false positive rate of P=0.05 using a binomial test as described.[8] Several exceptions to the rules to define putative somatic driver mutations were made in order to capture all relevant variants in known driver genes: (i) MAF >0.01 in UKB for *DNMT3A* R882, *JAK2* V617F and *GNB1* K57E; (ii) *TP53*: all mutations seen in at least once in COSMIC and validated in the International Agency for Research on Cancer database[9] were included (iii) *TET2*: all missense mutations were included in regions encoding the catalytic domains (amino acids 1104-1481 and 1843- 2002)[10; 11]; (iv) any *DNMT3A* variant was included that was seen at least once in COSMIC; (v) all frameshift indels, stopgain, and splice site mutations listed in a list of known myeloid neoplasia related genes were included[12].

Exceptions to the binomial test were made for established driver variants with high fitness, e.g. *U2AF1* Q157, *FLT3* Y842C, *JAK3* R657Q, *IDH2* R140L, *CBL* Y371H, and *KRAS* G12V due to the high fitness of these variants.[13] Variants that were absent from COSMIC were only considered if they had a heterozygous “0/1” or homozygous “1/1” genotype indicative of high quality genotype calls.

Diagnosis of ESKD

ICD10 codes used to classify ESKD in UKB participants were E85.3, N16.5, N18.0, N18.5, Q60.1, T82.4, T86.1, Y60.2, Y61.2, Y62.2, Y84.1, Z49.0, Z49.1, Z49.2, Z94.0, Z99.2. ESKD was also inferred by relevant interventions and procedures (OPCS4: L74.1, L74.2, L74.3, L74.4, L74.5, L74.6, L74.8, L74.9, M01.2, M01.3, M01.4, M01.5, M01.8, M01.9, M02.3, M08.4, M17.2, M17.4, M17.8, M17.9, X40.1, X40.2, X40.3, X40.4, X40.5, X40.6, X40.7, X40.8, X40.9, X41.1, X41.2, X41.8, X41.9, X42.1, X42.8, X42.9, X43.1)[14]

Prevalent and incident myeloid neoplasia

Participants with myeloid malignancy were identified from the national cancer registry and hospital inpatient records using the ICD10 codes C920, C921, C923, C924, C925, C927, C929, C930, C931, C940, C944, C946, C962, D45, D460, D461, D462, D464, D467, D469, D470, D471 and D473. Myeloid malignancies were considered prevalent if diagnosed before or within one year of study (n=320) entry, or incident (n=419) if diagnosed a year or more after study entry. The relationship between CH and ESKD in the absence of prevalent myeloid neoplasia was tested using multivariable logistic regression in R where ESKD diagnosed after the study entry was used as the dependant and CH as a binary predictor, and adjusted for the same CKD risk factors.

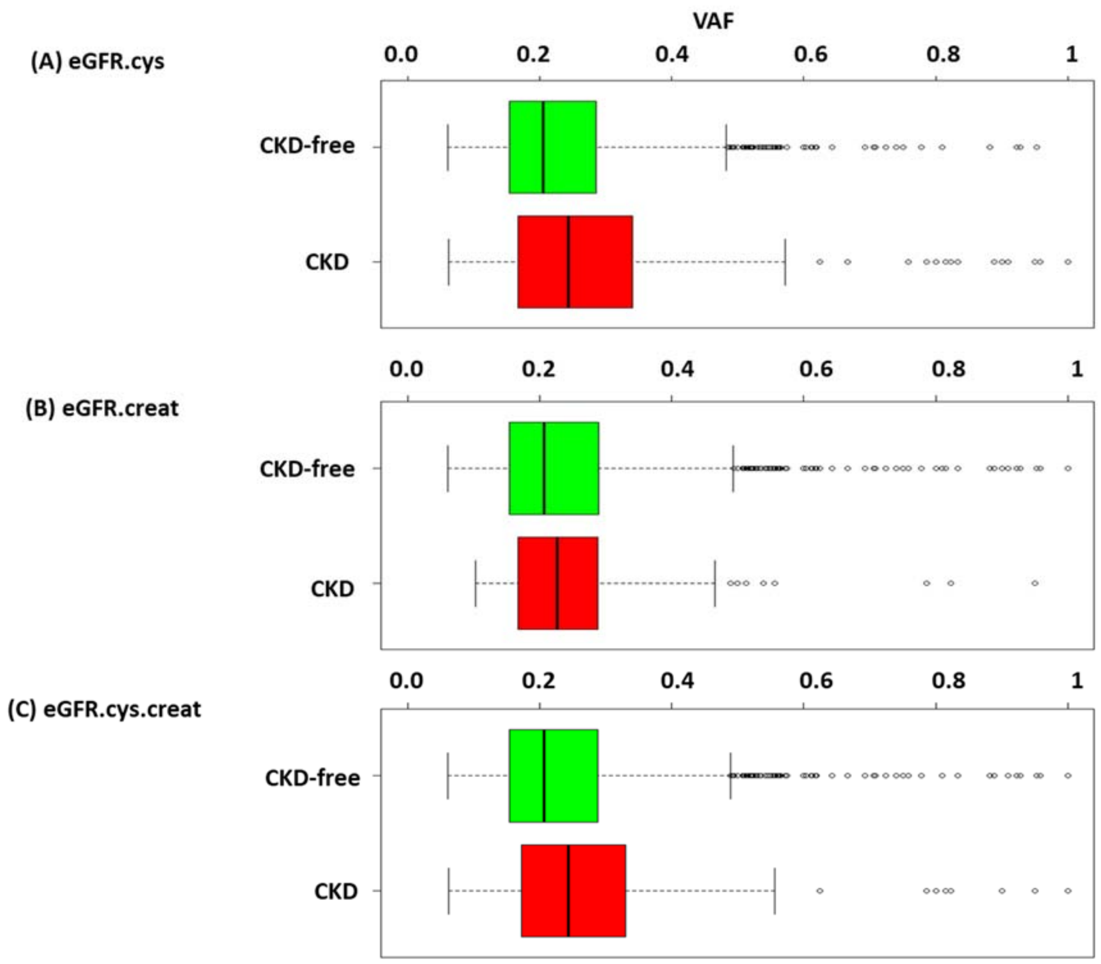

Supplementary Figure 1: CKD, defined by eGFR <60, is associated with VAF of driver mutations in myeloid related genes in 3,328 participants. Meta-analysis of discovery and validation cohorts (A) eGFR.cys: CKD (n=293), median = 0.24; CKD-free (n=3,035), median = 0.21 (P = $1.71x10^{-7}$; Mann-Whitney test), (B) eGFR.creat: CKD (n=111), median = 0.23; CKD-free (n= 3,217), median = 0.21 (P=0.12), (C) eGFR.creat.cys: CKD (n=144), median=0.23; CKD-free (n=3,184), median= 0.21 (P = 0.0002).

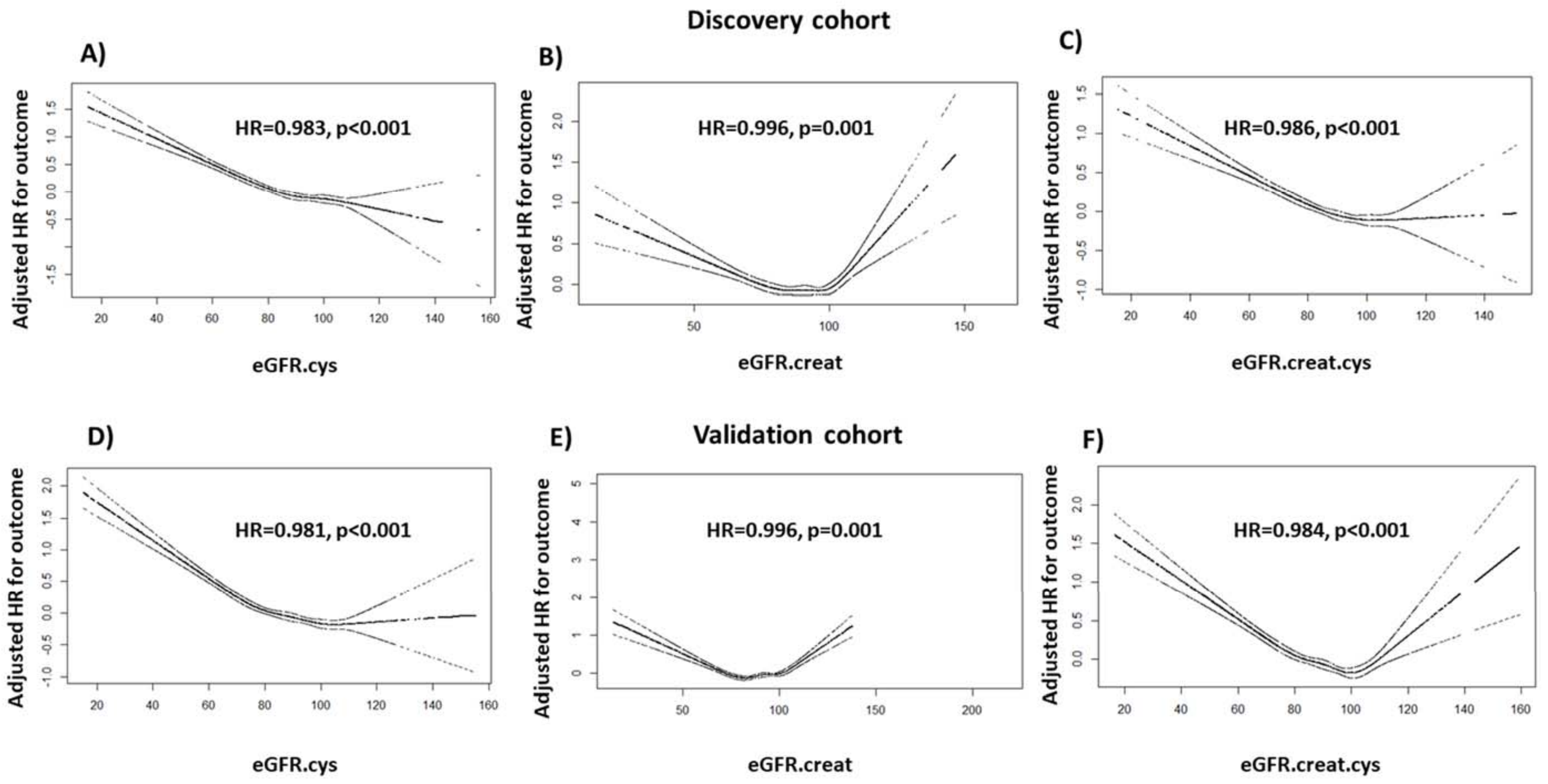

Supplementary Figure 2: Restricted cubic spline to test the linearity of eGFR scores. Adjusted spline of each eGFR score was plotted against HR for outcome with default values for the number of knots (n=5) and degrees of freedom (n=4). The upper and the lower dotted lines indicate 95% confidence intervals. A, B, and C refer to the discovery cohort. D, E, and F refer to the validation cohort for each eGFR score.

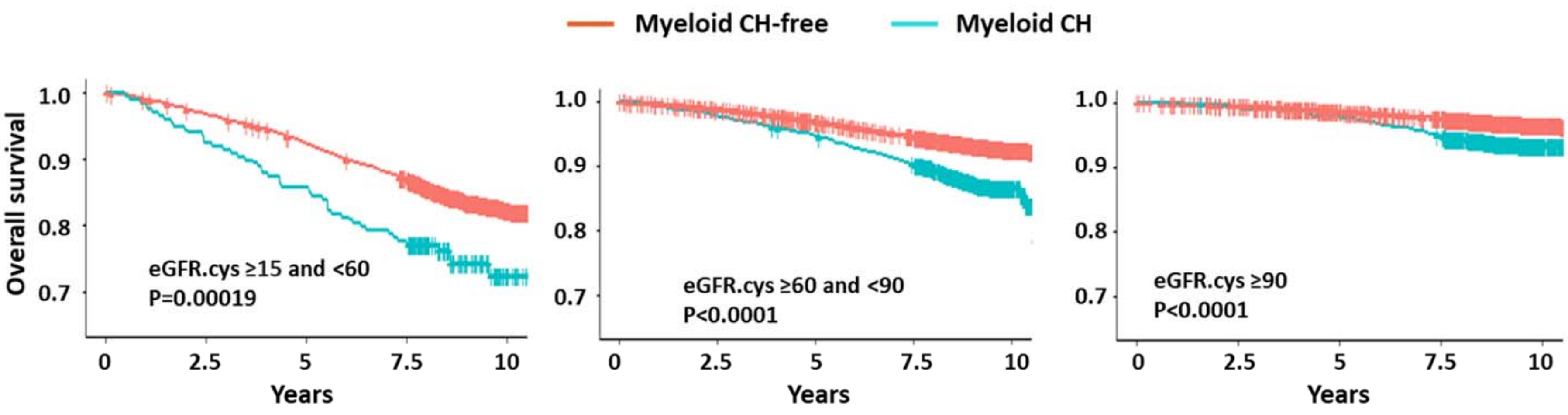

CH-free	6049	5844	5567	5234	481
CH	196	181	168	151	15

81320	80334	78679	76603	8157
1736	1696	1639	1563	148

88605	88137	87253	86109	10186
1116	1106	1089	1055	120

Supplementary Figure 3: Kaplan-Meier survival estimates for the three CKD groups according to absence or presence of myeloid CH, and excluding the 966 participants with potential SPS. Log-rank test P values are reported for each group, and numbers at risk at 0, 2.5, 5, 7.5, and 10 years after study entry.

Supplementary References:

1. Poplin, R., Chang, P.-C., Alexander, D., Schwartz, S., Colthurst, T., Ku, A., Newburger, D., Dijamco, J., Nguyen, N., and Afshar, P.T. (2018). A universal SNP and small-indel variant caller using deep neural networks. Nature Biotechnology 36, 983-987.
2. Li, H. (2011). A statistical framework for SNP calling, mutation discovery, association mapping and population genetical parameter estimation from sequencing data. Bioinformatics 27, 2987-2993.
3. Li, H., Handsaker, B., Wysoker, A., Fennell, T., Ruan, J., Homer, N., Marth, G., Abecasis, G., and Durbin, R. (2009). The sequence alignment/map format and SAMtools. Bioinformatics 25, 2078-2079.
4. Wang, K., Li, M., and Hakonarson, H. (2010). ANNOVAR: functional annotation of genetic variants from high-throughput sequencing data. Nucleic Acids Research 38, e164-e164.
5. Karczewski, K.J., Francioli, L.C., Tiao, G., Cummings, B.B., Alföldi, J., Wang, Q., Collins, R.L., Laricchia, K.M., Ganna, A., and Birnbaum, D.P. (2020). The mutational constraint spectrum quantified from variation in 141,456 humans. Nature 581, 434-443.
6. Kircher, M., Witten, D.M., Jain, P., O'Roak, B.J., Cooper, G.M., and Shendure, J. (2014). A general framework for estimating the relative pathogenicity of human genetic variants. Nature Genetics 46, 310-315.
7. Tate, J.G., Bamford, S., Jubb, H.C., Sondka, Z., Beare, D.M., Bindal, N., Boutselakis, H., Cole, C.G., Creatore, C., and Dawson, E. (2019). COSMIC: the catalogue of somatic mutations in cancer. Nucleic Acids Research 47, D941-D947.
8. Genovese, G., Kähler, A.K., Handsaker, R.E., Lindberg, J., Rose, S.A., Bakhoum, S.F., Chambert, K., Mick, E., Neale, B.M., and Fromer, M. (2014). Clonal hematopoiesis and blood-cancer risk inferred from blood DNA sequence. New England Journal of Medicine 371, 2477-2487.
9. Bouaoun, L., Sonkin, D., Ardin, M., Hollstein, M., Byrnes, G., Zavadil, J., and Olivier, M. (2016). TP53 variations in human cancers: new lessons from the IARC TP53 database and genomics data. Human Mutation 37, 865-876.
10. Hu, L., Li, Z., Cheng, J., Rao, Q., Gong, W., Liu, M., Shi, Y.G., Zhu, J., Wang, P., and Xu, Y. (2013). Crystal structure of TET2-DNA complex: insight into TET-mediated 5mC oxidation. Cell 155, 1545-1555.
11. Zhao, Z., Chen, S., Zhu, X., Pan, F., Li, R., Zhou, Y., Yuan, W., Ni, H., Yang, F.-C., and Xu, M. (2016). The catalytic activity of TET2 is essential for its myeloid malignancy-suppressive function in hematopoietic stem/progenitor cells. Leukemia 30, 1784-1788.
12. Jaiswal, S., Natarajan, P., Silver, A.J., Gibson, C.J., Bick, A.G., Shvartz, E., McConkey, M., Gupta, N., Gabriel, S., and Ardissino, D. (2017). Clonal hematopoiesis and risk of atherosclerotic cardiovascular disease. New England Journal of Medicine 377, 111-121.
13. Watson, C.J., Papula, A., Poon, G.Y., Wong, W.H., Young, A.L., Druley, T.E., Fisher, D.S., and Blundell, J.R. (2020). The evolutionary dynamics and fitness landscape of clonal hematopoiesis. Science 367, 1449-1454.
14. Lees, J.S., Welsh, C.E., Celis-Morales, C.A., Mackay, D., Lewsey, J., Gray, S.R., Lyall, D.M., Cleland, J.G., Gill, J.M., and Jhund, P.S. (2019). Glomerular filtration rate by differing measures, albuminuria and prediction of cardiovascular disease, mortality and end-stage kidney disease. Nature Medicine 25, 1753-1760.
